# Acquired immunological imbalance after surgery with cardiopulmonary bypass due to epigenetic over-activation of PU.1/M-CSF

**DOI:** 10.1186/s12967-018-1518-3

**Published:** 2018-05-25

**Authors:** Krzysztof Laudanski, Mateusz Zawadka, Jacek Polosak, Jaymin Modi, Matthew DiMeglio, Jacob Gutsche, Wilson Y. Szeto, Monika Puzianowska-Kuznicka

**Affiliations:** 1 Department of Anesthesiology, University of Pennsylvania, Philadelphia, PA 19146 USA; 2 Department of Anesthesiology, Medical University of Warsaw, Warsaw, 02-091 Poland; 3 Department of Human Epigenetics, Mossakowski Medical Research Centre, Warsaw, 02-106 Poland; 4 Philadelphia College of Osteopathic Medicine, Philadelphia, PA 19131 USA; 5 Division of Cardiovascular Surgery, University of Pennsylvania, Philadelphia, PA 19146 USA

**Keywords:** Epigenetics, Cardiopulmonary bypass, PU.1, Monocytes, Granulocyte colony stimulating factor

## Abstract

**Background:**

It has been shown that severe insult to the immune system may trigger prolonged macrophage characteristics associated with excessive release of monocyte colony stimulating factor (M-CSF). However, it is unclear how persistent is the macrophage-like characteristics in circulating monocytes (MO). In this study, 20 patients who underwent non-emergent cardiopulmonary bypass had their monocytes characterized before surgery and 3 months after surgery.

**Methods:**

We assessed the macrophage characteristics of MO using cytokine production, surface marker expression, an ability to stimulate T cells, and methylation of the promoter region of the gene encoding PU.1, a critical component to M-CSF production. MO function as well as activation and differentiation potential were longitudinally assessed.

**Results:**

At 3 months after cardiopulmonary bypass, monocytes exhibited increased expression of MRP8, transforming growth factor-β/latency-associated peptide, suppressor of cytokine signaling 3 while phagocytic properties were increased. Concomitantly, we observed a decreased expression of CD86, a decreased ability to form regulatory dendritic cells, and a diminished ability to stimulate T cells. These characteristics were accompanied by a persistent increase in the secretion of M-CSF, over-activation of PU.1, and decreased methylation of the PU.1 promoter region. Serum levels of C-reactive protein and anti-cytomegalovirus IgG antibody titers were also elevated in some patients at 3 months after surgery.

**Conclusions:**

We concluded that at 3 months after cardiopulmonary bypass, monocytes continued to express a new macrophage-like milieu that was associated with the persistent activation of the PU.1/M-CSF pathway.

## Background

Systemic inflammatory response syndrome (SIRS) after cardiopulmonary bypass procedure (CPB) broadly resembles the inflammatory responses seen in critical care illnesses and the postoperative period of extensive surgeries [1–6]. Moreover, the increase in immunoaberrance related morbidities has been shown to persist for many years after the initial insult, suggesting the acquisition of altered long-term immunostasis during severe insults [6–9].

Monocyte (MO) abnormalities are linked to susceptibility to various infections, the resurgence of opportunistic diseases, accelerated atherosclerosis, and premature failure of grafted coronary vessels during recovery from critical illness [1–3, 10–14]. Some attribute the aberrant MO performance to the persevered acquisition of new characteristics, but the evidence regarding the duration of these characteristics are missing. Moreover, heterogeneity of human MO is difficult to interpret in clinical settings due to lack of absolute flow cytometric markers, diversity in function and differentiation, and interspecies differences [15, 16]. A variety of MO subpopulations have been described such as steady-state, naïve, activated, patrolling, tolerogenic, alternatively activated, and atypical [11, 13, 17–20]. Functional plasticity allows MO to become M1 macrophages (MΦ) [typically CD16^(high)^, MRP^(high)^], or anti-inflammatory M2 cells [typically CD206^(high)^] [11, 12]. Alternatively, a small yet critical number of MO-derived dendritic cells (DC) emerge (MO → DC) quickly in critical care illnesses and facilitate the conversion of initial innate responses into acquired immunity and healing [17, 19, 21]. The emergence of MO subtypes depends on environmental influences, kinases (e.g., suppressor of cytokine signaling [SOCS]), and transcription factors (e.g., PU.1) [11–13, 15, 16]. The MO heterogeneity is the key to their ability to adapt and optimally respond to the challenges. An imbalance of emerging MO subtypes immediately post-insult skews the inflammatory response and increases morbidity [8, 14, 19–27]. For example, the inadequate emergence of DC from naïve MO results in the predominance of MΦ and overwhelming inflammatory response in trauma and burn patients [20–22, 25–27]. Conversely, the excessive emergence of M2, tolerogenic MO, or atypical MO leads to immunosuppression and increases the chance of infections, including cytomegalovirus (CMV) [13, 20, 21]. In patients undergoing cardiac surgery with CPB, the frequency of phagocytic MO is elevated shortly afterward, but an increase in MΦ-like MO may persist [CD64^(high)^ and CD32^(high)^] for 7 days [28]. This suggests that MO can be overcommitted developmentally [29]. Similar findings were observed in patients suffering from critical care insults, trauma, and burns but not a clear timeline for restoration of the immune system to pre-insult was ever defined. The excessive secretion of M-CSF is observed after acute inflammation, and prolonged M-CSF influence is related to MΦ predominance and other immune-aberrancies [13, 30, 31]. Continued M-CSF secretion can be sustained via a positive feedback loop involving either M-CSF and the M-CSF receptor (M-CSFR) or the activation of PU.1 [31, 32]. Consequently, we set up on investigating the activation of PU.1 3 months after surgery as the measure of recovery.

The major goal of this pilot study was to investigate the long-term characteristic of MO in the aftermath of CPB. We hypothesized that (1) CPB would result in the emergence of a new immunological balance in MO characteristics, (2) post-CPB MO abnormalities would be sustained by M-CSF secretion, secondary to PU.1 activation, and (3) these MO abnormalities might be associated with surrogates of unfavorable clinical outcomes.

## Methods

### Study population

The Institutional Review Board at the University of Pennsylvania (Philadelphia, PA) approved this study. The study was performed by the ethical standards in the 1964 Declaration of Helsinki and its later amendments. All participants in the study provided written informed consent.

We enrolled patients (n = 20) scheduled for non-emergency heart surgery with an application of CPB. The patients were recruited in two cohorts during the 2013 and 2014 calendar years. The total number of patients was defined by our experiences from a trauma cohort showing approximately 40% of immuno-aberrancy using the ability of MO to become DC as primary outcome [22]. This study was designed as an exploratory study, so no single outcome was purposely identified.

We excluded patients with pre-existing aberrant immunity (e.g., steroid use, active neoplastic illness, active autoimmune or rheumatologic disorders, and organ transplants). The patients’ clinical characteristics are presented in Table 1.

**Table 1:** Baseline characteristics of the patients

Characteristics	
Age—year	68.8 ± 13.4
Sex—Male no.	11.0 (55%)
BMI (kg/m^2^)	29.2 ± 5.3
Preoperative WBC (10^9^/L)	7.1 ± 1.9
Anesthesia and surgery data	
Duration of anesthesia (min)	406.4 ± 116.2
Duration of surgery (min)	290.0 ± 109.9
Duration of CBP (min)	138.4 ± 74.4
Duration of X-clamp (min)	111.8 ± 55.2
Coronary artery bypass surgery no.	8 (40%)
Mitral valvuloplasty no.	6 (30%)
Aortic valve replacement no.	4 (20%)
Aortic aneurysm repair no.	2 (10%)
Transfusions	
During surgery	
Packed red blood cells (ml)	0 [0;450]
Fresh frozen plasma (ml)	0 [0;500]
Platelets (ml)	0 [0;300]
In 24 h post-surgery	
Packed red blood cells (ml)	0 [0;300]
Fresh frozen plasma (ml)	0 [0;0]
Platelets (ml)	0 [0;0]
Total crystalloid during surgery (ml)	2000 [500;4750]
ICU stay	
APACHE score at 1 h	20.2 ± 5.0
APACHE score at 24 h	12.7 ± 5.4
APACHE score at 48 h	12.2 ± 4.6
Comorbidities	
Chronic heart failure	6 (30%)
Connective tissue disease (non-active)	1 (5%)
Peripheral vascular disease	4 (20%)
Cerebrovascular disease	5 (25%)
Type 2 diabetes	4 (20%)
Liver disease	0 (0%)
AIDS	0 (0%)
COPD	1 (5%)
Any tumor (last 5 year)	1 (5%)
Renal disease moderate to severe	4 (20%)

We collected blood before surgery (t_0_; baseline) followed by three post-op draws including, early postoperative (t_+24h_), late postoperative (t_+7d_), and long-term (t_+3m_). 30 ml of blood was collected. Serum was stored at − 80 °C. Mononuclear cells (MNC) depleted of granulocytes were separated using the Ficoll gradient technique followed by use of a negative magnetic selection of MO with DynaBeads^®^ allowing for separation of untouched and unstimulated MO (Life Technologies; Grand Island, NY) [22]. The purity of the MO population was measured as greater than 80% of CD14^(positive)^/CD33^(high)^ cells.

### Flow cytometry

A total of 10^5^ cells were incubated with FACS media (PBS without Ca^2+^ or Mg^2+^ with 0.01% sodium azide and 1% FBS) enriched with human True Stain FcX™ (BioLegend; San Diego, CA) for 15 min at 4 °C in the dark. Then, the cells were incubated with the antibodies for 30 min at 4 °C in the dark with additional mixing at 15 min. The cells were washed twice in FACS media and re-suspended in 100 μl of 1% FlowFix (Polysciences; Warrington, PA). The following antibodies conjugated with fluorophores were employed: CD1a (HI149; BioLegend; San Diego, CA), CD14 (Tuk4; Invitrogen; Grand Island, NY), CD83 (HB15e; BD; San Jose, CA), CD206 (15-2; BioLegend; San Diego, CA), CD86 (clone IT2.2; BioLegend; San Diego, CA), MRP8 (clone MRP 1H9; BioLegend; San Diego, CA), TGFβ/LAP (clone TW4-2F8; BioLegend; San Diego, CA), and TLR4 (clone HTA125; BioLegend, San Diego, CA).

Using a commercial kit (ThermoFisher; Rockford, IL), some of the naive antibodies including αhSOCS1 (clone C204; IBL; Chicago, IL), αhSOCS3 (clone J192; IBL, Chicago, IL) and αhPU.1 (clone Spi-1; Santa Cruz Biotechnology, San Diego CA) were conjugated with fluorescent dye (Alexa515, Alexa575). Appropriate non-specific IgG was used as an isotype control. After permeabilization with Wash Buffer (BioLegend; San Diego, CA), 10^5^ cells were stained intracellularly.

Zymosan A S. cerevisiae*FITC BioParticles^®^ (Life Technologies; Grand Island, NY) was reconstituted at 20 mg/ml in PBS without Ca^2+^/Mg^2+^ and opsonized, as per the manufacturer’s recommendation. A total of 10^5^ cells were incubated in 0.5 μg/ml of zymosan for 30 min at 37 °C in 5% CO_2_. The negative control was incubated at 4 °C in 5% CO_2_. After the addition of 5 µl of 0.1% Alamar Blue to 100 µl of the suspension, cells were analyzed using flow cytometry to quantify the amount of phagocytized zymosan. Data points were collected as mean fluorescent intensity (MFI).

The cells were analyzed with an LSR™ (BD; San Jose, CA), or a FACSCalibur™ (BD; San Jose, CA). At least 10^4^ cells were collected for each assessment. Duplets or dead cells were excluded by gating with forwarding and side scatter. Fluorescence of the unstained cells was used as a reference point [22]. Data are reported as a percentage of positive cells (%) while receptor density is presented as mean fluorescent intensity (MFI) and qualitatively assessed as ^(low)^, ^(medium)^, ^(high)^.

### Generation of immature dendritic cells from peripheral MO

The functional plasticity of the patients’ MO population was assessed by generating immature DC. Fresh monocytes were incubated in X-VIVO 15™ Media with Gentamycin and Phenol Red (Lonza, Cohasset, MN) supplemented with human interleukin 4 (hIL-4; Peprotech, Rocky Hill, NJ) at 500 IU/ml and human granulocyte monocyte colony stimulation factor (hGM-CSF; Peprotech, Rocky Hill, NJ) 1000 IU/ml 1 at 37 °C 5% CO_2_ in the dark. On day 3, 50% of the X-VIVO15/10™ was replenished with fresh media along with 50% of the initial cytokine concentration. The cultures were terminated on day 5 [22].

### Monocyte stimulation

In order to characterize cytokine secretion, 2 × 10^5^ of isolated MO were incubated in a total of 180 µl of X-VIVO10 (BioWhittaker; Walkersville, MD) combined with 20 µl of Alamar Blue (Life Technologies; Grand Island, NY) with lipopolysaccharide (LPS; 0111: B4, cell culture tested, gamma-irradiated, Sigma-Aldrich; St. Louis, MO) at a final concentration of 100 µg/ml for 18 h [22]. After incubation, cells were spun down, and the supernatant was stored at − 80 °C.

### Mixed lymphocyte reaction

A one-way mixed lymphocyte reaction (MLR) was employed to assess functional ability of MO or IL-4, and GM-CSF stimulated MO, to induce T cell proliferation—an ultimate measurement of their performance. 2 × 10^4^ harvested cells (IL-4 and GM-CSF differentiated MO or peripheral blood MO) were added to 2 × 10^5^ allogeneic T cells and incubated with 20 μl of Alamar Blue (Life Technologies; Grand Island, NY) that was subsequently added. Controls for DC MLR consisted of stimulator cells alone, T cells alone, and media mixed with Alamar Blue alone. Controls for MO MLR consisted of T cells alone and media mixed with Alamar Blue alone. After 18 (DC MLR) or 48 (MO MLR) hours of incubation, the absorbance at 570 nm (with a reference filter at 630 nm) was measured to assess T cell proliferation using the Opsys MR (Thermo Laboratories; Philadelphia, PA) with Revelation software (Thermo Laboratories; Philadelphia, PA). An increase in the percentage of Alamar reduction correlates with T cell proliferation.

### Cytokine and serum marker measurements

The supernatant level of cytokines (TNFα, IL-6, IL-10, M-CSF) and the serum concentration of inflammatory markers (CRP, haptoglobin, α-macroglobulin, serum amyloid P component) were measured using a magnetic multiplex kit (Bio-Rad; Hercules, CA), as per the manufacturer’s protocol, and analyzed on the BioRad™ platform (Hercules, CA).

ELISA assays were performed for IgG αCMV, and IgM αCMV (Abcam; Cambridge, UK) following the manufacturer’s protocols. The data for IgM αCMV are reported as positive vs negative results (qualitative testing). The absorbance was measured using the Opsys MR (Thermo Laboratories; Philadelphia, PA) with Revelation software (Thermo Laboratories; Philadelphia, PA).

### Epigenetic analysis of PU.1/SPI1 methylation

To assess the epigenetic regulation of PU.1, an analysis of methylation of SPI1 was conducted. Using the CpG Island Finder program (http://dbcat.cgm.ntu.edu.tw), we analyzed 3 kb fragment of the *SPI1* gene encoding the PU.1 transcription factor. The analyzed fragment was located from 1.5 kb upstream to 1.5 kb downstream of the position corresponding to the first base of the major gene transcript (potential transcription initiation site). Within the 750 bp long fragment indicated as the CpG island, a 375 bp fragment containing 17 CpG dinucleotides of the potential transcription start sites of the major and two other gene transcripts, were selected for further analysis. The methylation status of this fragment was analyzed using the OneStep qMethyl Kit (Zymo Research; Irvine, CA) following the manufacturer’s protocol. The real-time PCR reaction was performed using LightCycler 480 II (Roche Diagnostics; Mannheim, Germany). The primers used were: forward 5′ATGTCACCCCAAGGGGACTA3′ and reverse 5′GGAAACCCTGACTTCCCACT3′. The PCR conditions were: initial denaturation for 10 min at 95 °C, then 45 cycles of 30 s at 95 °C, 30 s at 63 °C, 30 s at 72 °C, and then one melting curve cycle.

### Statistical analysis

The datasets generated and analyzed during the current study are available from the corresponding author upon request.

No a priori statistical power calculations were conducted, since this study was conducted as a pilot project. Using the prior data obtained from trauma patients showing a 40% frequency of MO aberration, we decided to study 20 patients total [22]. Blood samples were taken at the four different data points [baseline (t_0_), 24 h (t_+24h_), 7 days (t_+7d_), and 3 months (t_+3m_) from patients undergoing CPB. Data were always compared to the pre-CPB, baseline, the value of the same patient (t_0_).

Initially, a descriptive analysis of the data was performed. Data meeting the parametric assumption of normality and not distorted by the presence of outliers is presented in tables as a mean within one standard deviation. Data not meeting above assumptions is presented as median and 1st and 3rd quartiles. Normality was assessed using Shapiro–Wilk test and normal quantile plots. Single missing values in a data series (one of the markers not measured at either baseline, 24 h, 3 days, or 3 months as indicated in a given patient) are due to random errors of the measuring instruments and were filled using the *MICE* algorithm *(Multivariate Imputation by Chained Equations).* This equation derives missing values from the rest of the values using the *predictive mean matching* method, which is the average of 10 values computed in independent iterations of the *MICE* algorithm.

Analyses considered are either comparisons of two-time points or more than two-time points. In the former comparison, parametric data were analyzed using paired *t* test with 95% CI for the mean difference between the measurements. The effect size was assessed using paired Cohen’s *d* coefficient. Non-parametric data were analyzed using Wilcoxon signed rank test with 95% CI for the pseudo-median (Hodges–Lehman statistic) of differences. The effect size was assessed using *Common Language Effect Size* measure which in this case was a simple fraction of observation supporting a hypothesis saying that one measure dominates the other. In the case of multiple time points, Friedman test was performed. If significant, it was followed by a many-to-one Dunnet’s type *posthoc* testing procedure comparing only the baseline with the other measures (t_24h_, t_7d_, and t_3m_) so only *p* values for these comparisons were computed and adjusted. Magnitudes of differences between measurements were assessed on the ground of 95% CI for differences between measurements’ rank sums. Effect sizes were again assessed using *CLES* coefficient. Results of multiple testing, both in the case of post hoc comparisons after Friedman tests and several conceptually related two-sample tests, were adjusted using Benjamini–Hochberg FDR (*False Discovery Rate*). It was preferable to use this approach instead of more stringent FWER (*Family*-*Wise Error Rate*) due to the very limited sample size and the pilot character of the study which implies that higher power may be preferable even at the cost of slightly higher type I error. There was also one case of a comparison of two independent samples. The data was parametric in this case, so Welsch *t* test with 95% CI for the difference of the means was performed. The effect size was assessed using Cohen’s *d* coefficient.

## Results

**Fig. 1:**
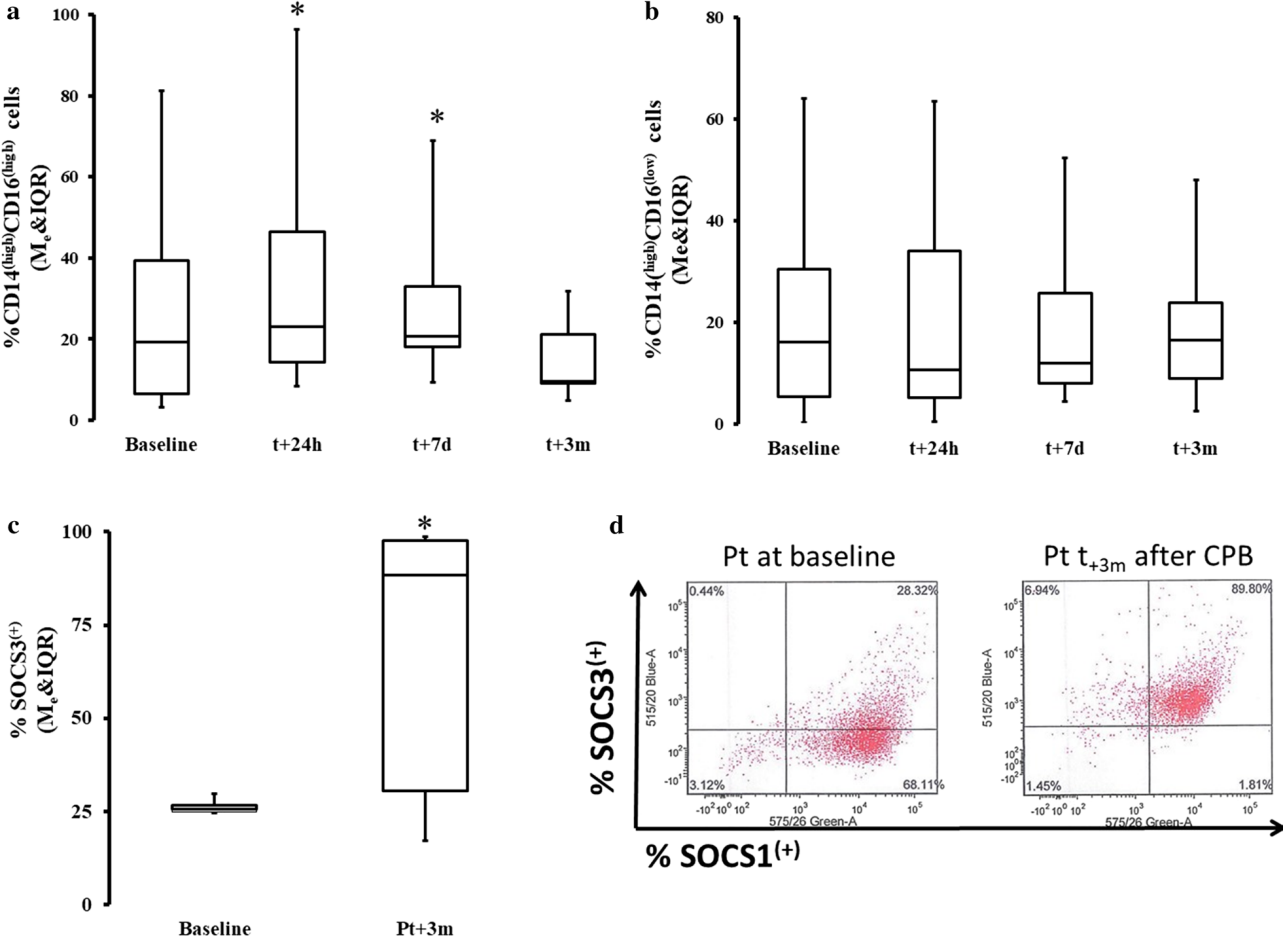
Change in the phenotype and kinase makeup of the post-CPB. Fresh MO were stained with flow cytometry antibodies. There was a significant difference in post hoc analysis baseline in frequency  %CD14^(high)^CD16^(high)^ MO (Me = 13.54; IQR[6.15, 24.39] ) vs t + 24 h and vs t + 7d, t_24h_: Me = 22.63; IQR[13.32, 45.06] *ΔRank* = − 15.00; *CI*_*95%*_ = [− 27.81, − 2.19]; *p* = 0.034; *CLES* = 0.86; t_7d_: Me = 20.58; IQR[18.11, 33.09]; *ΔRank* = − 15.00; *CI*_*95%*_ = [− 27.81, − 2.19]; *p* = 0.034; *CLES* = 0.71 (**a**). There was no significant difference in %CD14^(high)^CD16^(low)^ (**b**). A significant increase in predominantly M2-promoting kinases were seen in peripheral blood MO population at 3 months. The population SOCS3^(high)^ cells was significantly increase at t_3m_: Me = 88.46; IQR[30.56, 97.60] as compared to pre-CPB level: Me = 24.77; IQR[24.17, 25.83] ΔpMe = − 39,41; CI_95%_: ΔpMe[− 72.47, − 3.16]; p = 0.047; CLES = 0.78 (**c**). This shift is very clear if data from the same patient are shown (**d**). Asterisk indicate data points which were significantly different when compared vs baseline. *CI*_*95%*_: 95% confidence interval for difference in sum of ranks. *CLES* common language effect size

**Fig. 2:**
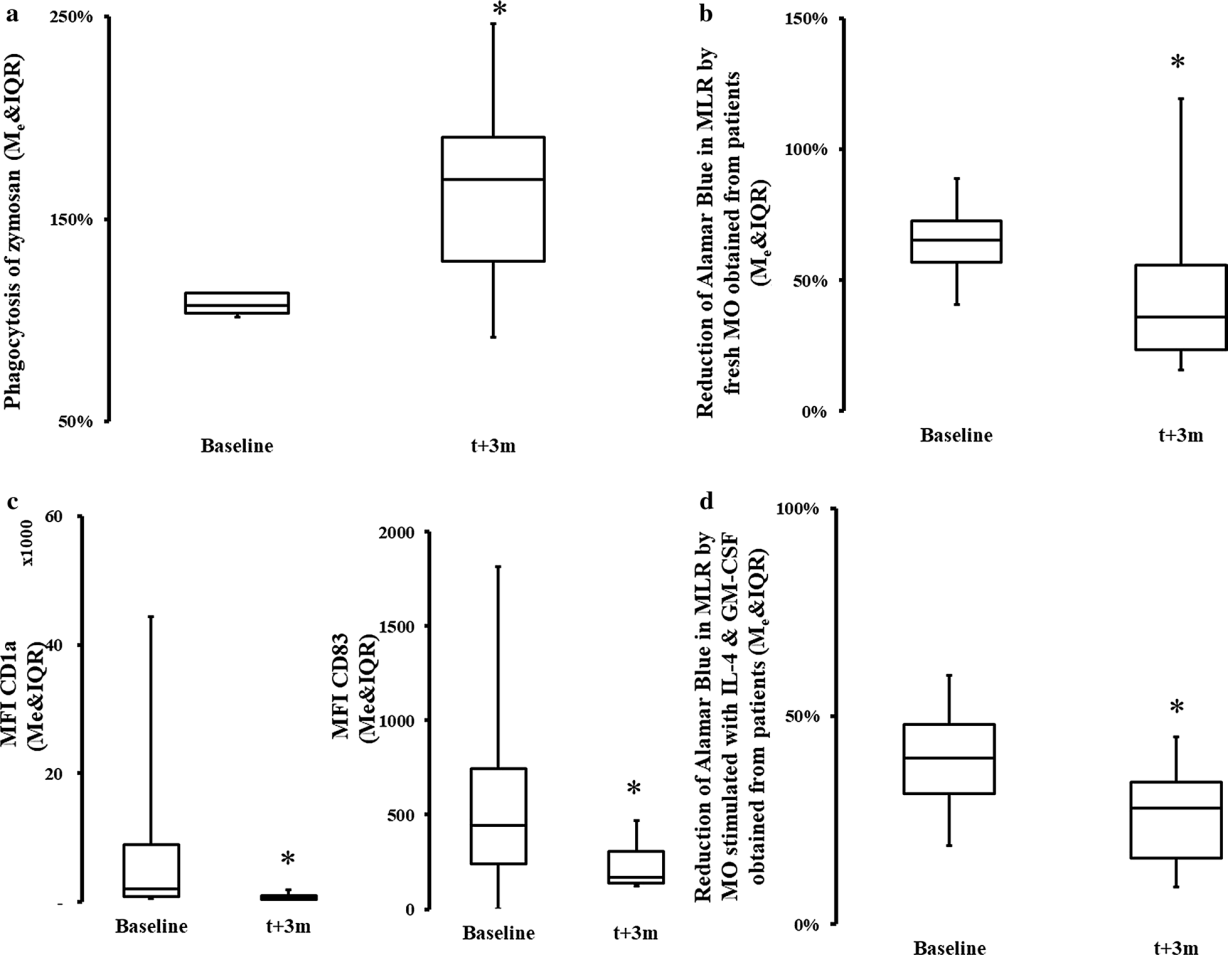
Changes in monocyte function after surgery. Functional properties of the circulating MO were tested using, phagocytosis assays (**a**), ability to stimulate allogeneic T cells (**b**), functional plasticity by incubate cells in vitro with IL-4 and GM-CSF and measuring an emergence of DC specific marker (**c**) or ability to induce proliferative T cells response. **a** There was a significant increase (t_0_: Mean = 10.7 ± 0.05 vs t_3m_: Mean = 1.64 ± 0.49 *CI*_*95%*_: Δx = [0.23, 0.90]; *p* = 0.003; *d* = 1.36) in the phagocytic capabilities of peripheral blood monocytes taken from patients at 3 months after the surgery, as shown by uptake of zymosan. **b** The ability of peripheral blood monocytes to stimulate allogeneic T cells was significantly (t_0_: Mean = 0.65 ± 0.13 vs t_3m_: Mean = 0.46 ± 0.31; *CI*_*95%*_ Δx [− 0.33, − 0.04]; *p* = 0.016; *d* = − 0.68) diminished at 3 months. **c** There was a significant decline in the emergence of CD1a^(+)^ (immature DC marker) (t_0_: Me = 1964.58; IQR[745.82, 7586.04] vs t_3m_: Me = 519.19; IQR[428.24, 785.52]; CI_95%_: ΔpMe[761.49, 5459.51]; p = < 0.001; CLES = 1.0) and CD83 (mature DC marker) (t_0_: Me = 64.72; IQR[43.22, 82.55] vs t_3m_: Me = 445.24; IQR[240.07, 747.04]; CI_95%_: ΔpMe [101.62, 964.67]; p = 0.047; CLES = 0.75). **d** Stimulation with IL-4 and GM-CSF did not recover MO ability to stimulate T cells to pre-CPB levels (t_0_: Me = 39.50; IQR[30.75, 48.50] vs t_3m_: Me = 26.40; IQR[18.25, 34.00]; *ΔRank* = 21.50; *CI*_*95%*_ = [6.83, 36.17]; *p* = 0.015; *CLES* = 0.83). Asterisk indicate data points which were significantly different when compared vs baseline. *CI*_*95%*_: Δx-95% confidence interval for difference in means. *CI*_*95%*_: 95% confidence interval for difference in sum of ranks. CI_95%_: ΔpMe-95% confidence interval for difference in pseudomedians

In the first step, we evaluated characteristics of peripheral blood MO using flow cytometric markers and functional assays to define their developmental characteristics 3 months post-CPB. We found a significant increase in inflammatory CD16^(high)^ MO, but only immediately after surgery (Fig. 1a). At 3 months, the frequency of CD14^(high)^CD16^(high)^ MO returned to pre-CPB levels (Fig. 1a). We also found that the MO population had increased surface density of CD206, CD163 and TGFβ/LAP while expression of CD86 was diminished (Table 2). A significant increase in SOCS3 positive cells was observed while SOCS1 remained more closely to the pre-CPB level at 3 months post CPB (Fig. 1c, d, Table 2). There was also a significant increase in the phagocytic capacity of the peripheral blood MO obtained 3 months after surgery (which were pre-incubated with zymosan) as compared to the performance of MO before CPB (Fig. 2a). Moreover, the ability of peripheral blood MO to stimulate allogeneic T cells was severely diminished at 3 months after surgery (Fig. 2b). Concomitantly, we noticed that there was a significant decline in the emergence of CD1a^(+)^ (immature DC marker) and CD83^(+)^ (mature DC marker) on IL-4 and GM-CSF-differentiated MO even 3 months post-surgery in the CPB cells (Fig. 2c) [12, 13, 20]. IL-4 and GM-CSF-differentiated MO had a significantly depressed ability to stimulate T cells in MLR if the MO were obtained from subject 3 months post-CPB compared to pre-CPB levels (Fig. 2d). The frequency of endogenous BDCA-3/CD11c(+) dendritic cells was also diminished at 3 months post-surgery (%BDCA-3t_0_ = 2.14 ± 3.08 vs %BDCA3t_+3m_ = 0.55 ± .63; p < 0.05).

**Table 2:** Flow cytometric landscape of the MO 3 months after CPB compared to pre-CPB levels

Marker	Typical cellular expression	t_o_ (M_e_ [IQ_25_; IQ_75_])	t_3m_ (M_e_ [IQ_25_; IQ_75_])	*p*
MFI-MRP8	MAC M1	2372.33 [1250.74, 2977.14]	774.4 [695.33, 1032.22]	0.019
MFI CD86	Activated MO	783.88 [457.60, 990.84]	230.17 [178.29, 372.42]	0.050
MFI-CD206	MAC M2	725.00 [409.81, 1038.23]	1902.2 [905.26, 4212.51]	0.026
MFI-CD163	MAC M1	1653.80 [1400.21, 1885.59]	1335.6 [718.19, 1439.18]	0.004
TGF/LAP	MAC M2	1099.53 [892.44, 1253.50]	3223 [2407.00, 3560.50]	0.003

Flow cytometry reveals a persistence of MRP^(high)^, CD206^(high)^, CD163^(high)^, TGF/LAP^(high)^ and decreased expression of CD86 3 months after CPB as compared to pre-CPB values

**Fig. 3:**
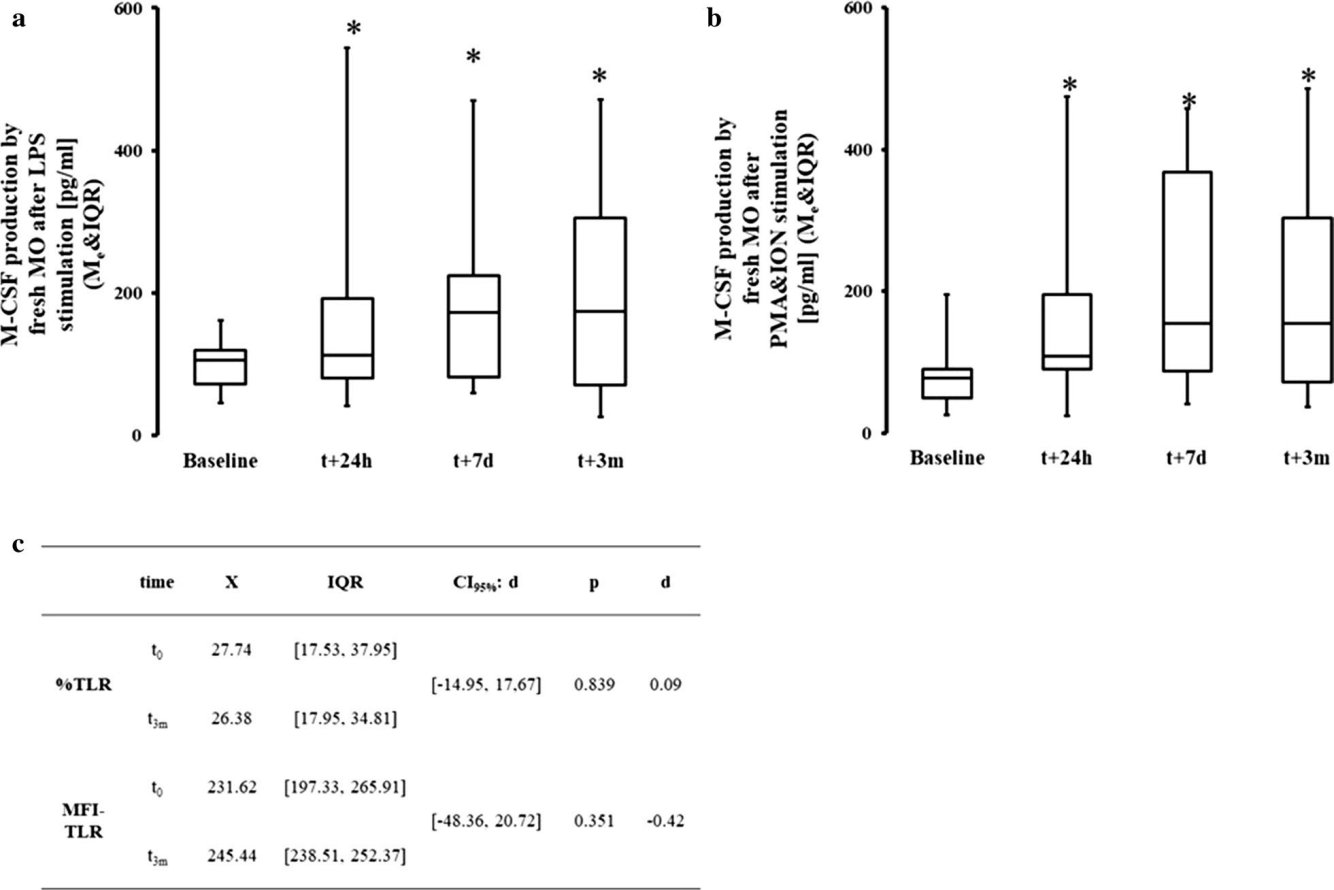
Changes in post-CPB M-CSF production. Production of M-CSF in response to LPS (**a**) and PMA and ION (**b**) was assessed in vitro together with flow cytometric expression of M-CSFR and TLR on circulating MO (**c**). **a** There was a significant difference in all data points in M-CSF production with LPS stimulation t_0_: Me = 121.00; IQR[48.50, 189.00] t_24h_: Me = 4567.00; IQR[1298.50, 5755.00]; *ΔRank* = − 31.00; *CI*_*95%*_ = [− 44.28, − 17.72]; *p* = 0.003; *CLES* = 0.89; t_7d_: Me = 2053.00; IQR[375.50, 7036.50]; *ΔRank* = − 30.00; *CI*_*95%*_ = [− 43.28, − 16.72]; *p* < 0.001; *CLES* = 0.89; t_3m_: Me = 1346.00; IQR[192.00,5627.00] *ΔRank* = − 21.00; *CI*_*95%*_ = [− 34.28, − 7.72]; *p* = 0.003; *CLES* = 0.79. **b** Production of M-CSF after PMA and ION stimulation was increased at all data point when compared with baseline t_0_: Me = 93.00; IQR[38.00, 192.50]; t_24h_: Me = 3321.00; IQR[530.00, 6532.00]; *ΔRank* = − 22.00; *CI*_*95%*_ = [− 34.73, − 9.27]; *p* = 0.002; *CLES* = 0.94; t_7d_: Me = 2341.00; IQR[265.00, 8356.50]; *ΔRank* = − 21.00; *CI*_*95%*_ = [− 33.73, − 8.27]; *p* = 0.002; *CLES* = 0.69; t_3m_: Me = 341.00; IQR[106.00, 4249.00]; *ΔRank* = − 23.00; *CI*_*95%*_ = [− 35.73, − 10.27]; *p* = 0.002; *CLES* = 0.88. **c** Receptor expression of TLR4 was unchanged in terms of both positive cells and receptor density (MFI). Asterisk indicate data points which were significantly different when compared vs baseline. *CI*_*95%*_: 95% confidence interval for difference in sum of ranks. CI_95%_: ΔpMe-95% confidence interval for difference in pseudomedians. *CLES* common language effect size

We found a significant increase in the secretion of M-CSF in response to LPS in supernatants of MO both immediately following and 3 months after surgery (Fig. 3a). A similar trend was seen after stimulation of MO by PMA and ION (Fig. 3b). In contrast, no persistent increase in secretion of IL-1β, IL-6, or IL-10 was seen at 3 months post-CPB after stimulation with LPS (Table 3). The elevated secretion of M-CSF is not due to an increased sensitivity to LPS signals since the surface expression of TLR4 remained unchanged before and after CPB (Fig. 3c).

**Table 3:** Cytokine secretion by peripheral monocytes

Cytokine (stimulant)	Baseline		3 months after CPB	
	Mean	SD	Mean	SD
IL-1β (LPS)	23,886.4	26,986.1	13,108.4	10,949.3
IL-1β (P&I)	7196.9	4388.7	8363.2	6840.8
IL-6 (LPS)	101,496.5	19,494.2	56,470.2	47,780.5
IL-6 (P&I)	38,233.9	12,750.2	52,475.5	41,079.9
IL-10 (LPS)	57,437.3	65,227.8	13,228.6	16,455.6
IL-10 (P&I)	4948.5	2698.2	11,942.9	9126.7
TNFα (LPS)	127,605.4	56,909.5	89,928.2	67,496.8
TNFα (P&I)	33,291.7	15,980.3	67,759.4	59,473.3

Secretion of IL-1β, IL-6, IL-10, and TNFα by peripheral blood monocytes was not significantly different 3 months after CPB in response to either LPS or combination of PMA and ION (stimulant denoted by parentheses).

**Fig. 4:**
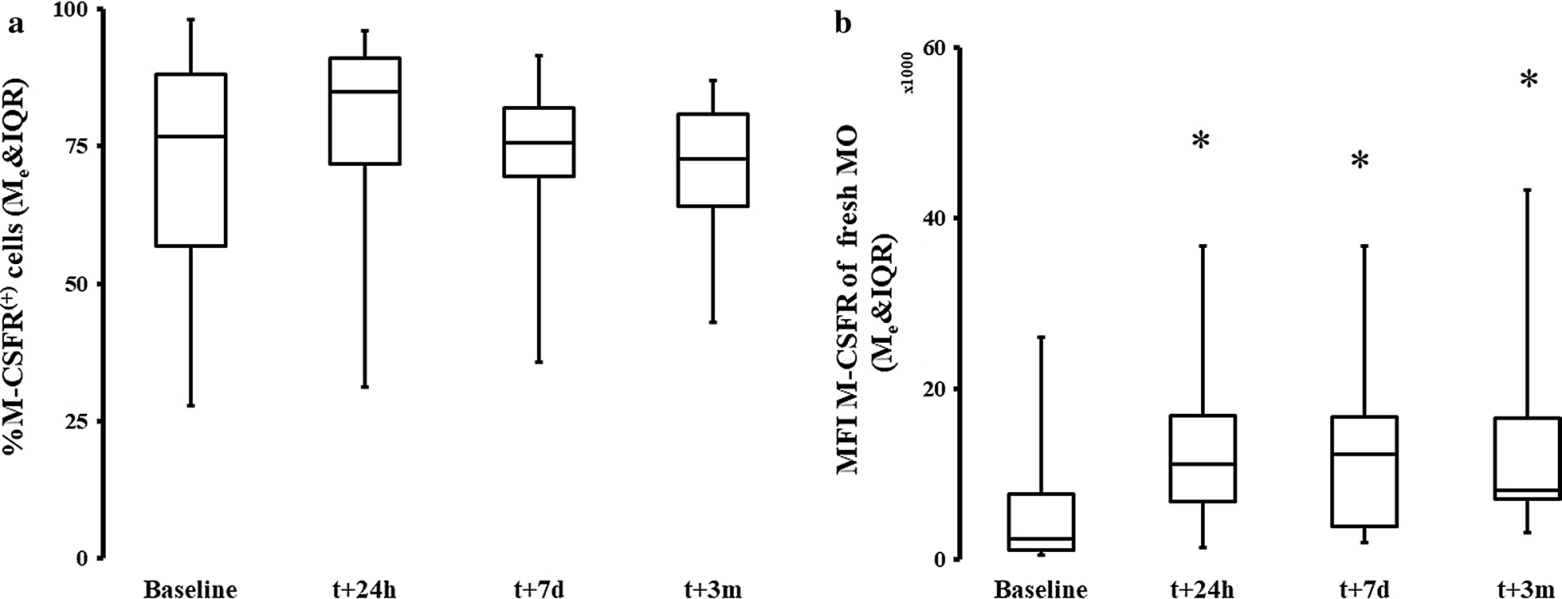
Expression of M-CSF receptor significantly increases and persists after CPB. **a** The expression of M-CSF receptors in terms of positive cells was not significantly different in a group analysis on the surface of circulating MO. **b** The CD115/M-CSFR receptor density was significantly increased at all times vs baseline after the surgery t_0_: Me = 2314.64; IQR[1032.07, 4978.19] t_24h_: Me = 10,425.65; IQR[8041.68, 16,667.99]; *ΔRank* = − 22.50; *CI*_*95%*_ = [− 35.59, − 9.41]; *p* = 0.002; *CLES* = 0.82; t_7d_: Me = 12,824.02; IQR[4165.53, 16,443.61]; *ΔRank* = − 18.5; *CI*_*95%*_ = [− 31.59, − 5.41]; *p* = 0.007; *CLES* = 0.82; t_3m_: Me = 9858.70; IQR[7541.30, 16,547.60] *ΔRank* = − 25.00; *CI*_*95%*_ = [− 38.09, − 11.91]; *p* = 0.001; *CLES* = 0.82. Asterisk indicate data points which were significantly different when compared vs baseline. *CI*_*95%*_: 95% confidence interval for difference in sum of ranks. CLES: common language effect size

**Fig. 5:**
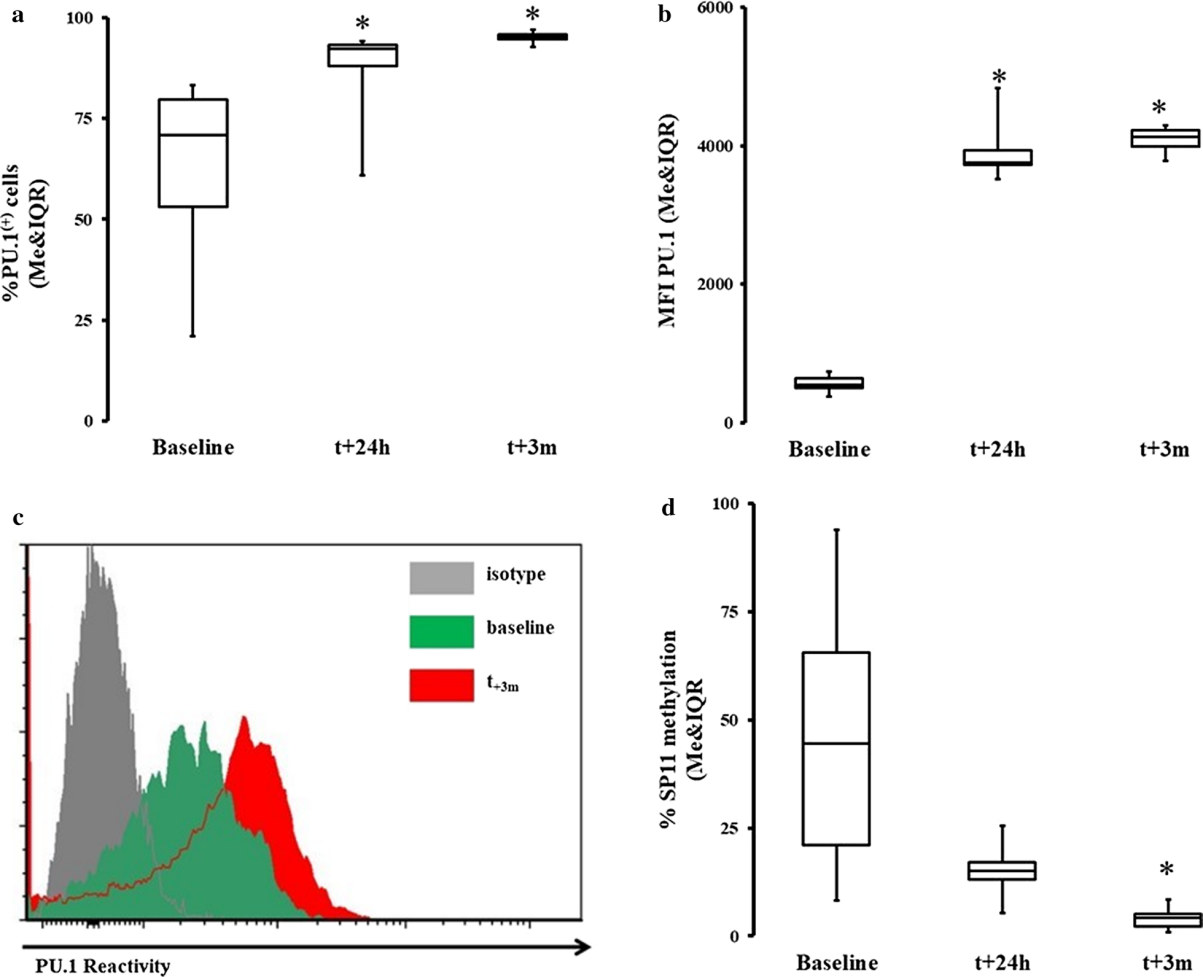
Increase in PU.1 activity coupled with epigenetic regulation of its gene promoter. Intracellular expression of PU.1 and methylation of SPI were measured in the circulating MO. **a** There was a significant increase in the percentage of expression of PU.1 t_0_: Me = 70.76; IQR[53.16, 79.72] t_24h_: Me = 90.14; IQR[83.88, 92.94]; *ΔRank* = − 4.00; *CI*_*95%*_ = [− 7.15, − 0.85]; *p* = 0.018; *CLES* = 0.83; t_3m_: Me = 94.99; IQR[94.47, 95.68]; *ΔRank* = − 11.00; *CI*_*95%*_ = [− 14.15, − 7.85]; *p* < 0.001; *CLES* = 1.00. **b** There was also a significant increase in antigen density (MFI) at all data points t_0_: Me = 545.30; IQR[500.20, 638.62]; t_24h_: Me = 3839.20; IQR[3729.39, 3944.17]; *ΔRank* = − 7.00; *CI*_*95%*_ = [− 10.15, − 3.85]; *p* = 0.001; *CLES* = 1.00; t_3m_: Me = 4129.11; IQR[3993.60, 4222.01]; *ΔRank* = − 11.00; *CI*_*95%*_ = [− 14.15, − 7.85]; *p* < 0.001; *CLES* = 1.00. **c** Again, this pattern was demonstrated on the MO obtained from the same patient (grayed graph is isotype control and auto fluorescence, green is baseline while red represent patient MO PU.1 reactivity at 3 months after CPB). **d** There was a significant decrease in methylation of SP11 gene at 3 months t_0_: Me = 49.65; IQR[39.50, 70.71]; t_3m_: Me = 4.61; IQR[4.27, 5.56]; *ΔRank* = 12.00; *CI*_*95%*_ = [5.71, 18.29]; *p* = 0.004; *CLES* = 1.00. Asterisk indicate data points which were significantly different when compared vs baseline. *CI*_*95%*_: 95% confidence interval for difference in sum of ranks. CI_95%_: ΔpMe-95% confidence interval for difference in pseudomedians. CLES: common language effect size

M-CSF is a cytokine induced by several pathways, but most potently via activation of CD115 (M-CSFR) followed by PU.1 [30, 31]. We found that the percentage of CD115^+^ positive cells were similar at all measured time points (Fig. 4a). M-CSF-R/CD115 surface density for M-CSF was significantly increased shortly after and 3 months after surgery (Fig. 4b). We also found an increase in the frequency of PU.1 positive MO as compared to pre-CPB levels (Fig. 5a–c). Finally, we found that there was a significant decrease in the methylation of the *SPI1* promoter fragment flanking the potential transcription start site of the three transcripts (main PU.1 transcript and two other transcripts) at 3 months post-CPB (Fig. 5d).

No clinically-relevant complications (e.g., incidence of sepsis, pneumonia, sternal wound infection, or acute kidney injury) could be appreciated in our study due to the low frequency and pilot nature of the study. Therefore, we focused on surrogates of abnormal function in the immune system. C-reactive protein (CRP), but not other inflammatory markers, were elevated at 3 months but the heterogeneity of the response was apparent (Table 4). Interestingly, the M-CSF production by MO upon stimulation with LPS correlated with serum level of CRP (*r*^*2*^ = 0.82; *p* = 0.0062) and SAP (*r*^*2*^ = 0.61; *p* = 0.026). Furthermore, the titer of IgG αCMV was increased in our patient population 3 months after surgery (IgG αCMV_t0_ = 2.54 ± 1.88 vs IgG αCMV_t+3m_ = 5.27 ± 2.56; Δx = − 2.73; *CI*_*95%*_ [− 5,32; − 0,13]; p = 0.041; d = − 1.71). The titer of IgM αCMV was unchanged at 3 months after surgery as compared to the baseline (data not shown since the test is qualitative).

**Table 4:** Serum levels of several inflammatory markers

Inflammatory marker	Baseline		3 months after CPB	
	Mean	SD	Mean	SD
C reactive protein (pg/ml)	224	587	1858*	2588
Alpha-2-macroglobulin (pg/ml)	95	69	363	921
Haptoglobin (pg/ml)	73	111	191	314
Serum amyloid P component (pg/ml)	28	30	64	80

Serum levels of inflammatory were not elevated 3 months after CPB except C-reactive protein

* Indicate data points which were significantly different when compared vs baseline in post hoc analysis. There was a significant increase in the percentage of expression of serum CRP at t_0_ vs t_+3m_ (p < 0.042)

## Discussion

This pilot study aimed to provide evidence of a remodeled post-CPB MO milieu 3 months after surgery and elucidate the potential role of the M-CSF/PU.1 system its persistence. Our study shows acquisition of a new MO balance, which was characterized by an increase in MRP8^(high)^, TGFβ/LAP^(high)^, CD115^(high)^, and SOCS3^(high)^, as well as augmented phagocytic capabilities for at least 3 months after CPB. Decreases in the expression of CD86, the ability of MO to become DC, and T-cell stimulatory capacity were also observed. HLA-DR expression on MO was recovering already at 7 days after CPB but these data are of pilot nature (data not shown). We concluded that 3 months after CPB, peripheral blood MO resemble both MΦ and M2 cells [11, 12, 22]. These characteristics are different from classical or nonclassical MO. We expanded prior research by looking much further after the initial insult and seeking the potential mechanisms [20, 22–24]. Furthermore, we explored the possible mechanisms involved in sustaining the post-CPB MO milieu. Post-CPB MO produced a large amount of M-CSF, whereas the secretion of other cytokines returned to baseline levels. We demonstrated that positive feedback between M-CSF secretion and M-CSFR is present. Most notably, the overexpression of PU.1, a master factor in the stimulation of M-CSF, was demonstrated at 3 months after CPB [31]. The methylation of the promoter region encoding PU.1 was decreased, suggesting a potential mechanism of post-CPB persistence of acquired characteristics in MO. Finally, we attempted to link the emergence of clinically relevant surrogates of morbidity in our patients despite study not being intended for looking for clinical variables. We noticed signs of immuno-incompetency (increase in serum αCMV IgG) and smoldering inflammation (increase in CRP in some subjects) [33–35]. Elevation in CRP was highly correlating with the M-CSF production by MO. The small sample size and observed heterogeneity of the inflammatory markers 3 months post-CPB, warrants a larger and more focused study.

The process of immune system response has distinctive time evolution. The immediate innate response is modulated by circulatory MO (among others) [5, 6, 36]. At later stages, acquired immunity becomes dominant, and DC are pivotal regulators [14, 19, 21]. Inflammation is then extinguished via the compensatory anti-inflammatory response syndrome. Along with this trajectory, the MO population characteristics change over time, and their plasticity is critical. Under optimal conditions, the inflammatory MO emerge quickly from peripheral blood MO and are replaced by tissue repair MΦ and other components of acquired immunity, but the exact duration of this process is unclear in subjects recovering from surgery or critical care illness [6, 11, 13]. In our study, we attempted to characterize the MO population at 3 months after surgery at the time when most patients are in recovery stages. We noticed that flow cytometric and functional features of circulating MO resembled deactivated MO, MΦ, and M2 cells [11, 13]. Persistent expression of SOCS3 and SOCS1 at 3 months suggests persistence of both the M2 and M1 phenotype respectively [11, 12, 32, 36]. An increased ability to phagocytose, a decreased ability to become dendritic cells, and the poor ability to circulate MO to stimulate T cells highly suggest that circulating MO resemble deactivated MΦ more than activated MO [11, 13, 22]. For future research, we into to employ RNA-seq to more definitely establish MO features considering that transcriptional markers are more definitive, though more costly than flow cytometry markers [12, 31, 32]. We demonstrated that certain newly-acquired characteristics of MO persisted over a 3-month period, which is well in the recovery period, in contrast to other short-term studies [2, 24, 26].

At 3 months acute inflammation should resolve, and MO should participate in tissue healing. Cytokines, especially M-CSF, modulate MO differentiation and function [11–13]. In contrast to other immune-inhibitory cytokines, M-CSF secretion is supported by a positive feedback loop [30, 37–39]. This positive feedback loop relies on PU.1 activation by secreted M-CSF and can reduce the ability of MO to stimulate T cells, trigger increases in atherosclerotic markers, inhibit the MO → DC process, augment the MO → MΦ process, and directly induce immune anergy [22, 30, 34, 36, 40]. Thus, M-CSF is one potential mechanism for long-term, post-critical care insult immunosuppression [6, 36]. We observed increased secretion of M-CSF but no other cytokines 3 months after surgery, suggesting a reprogramming of cells in their response to pathogen pattern recognition [6, 7, 36]. This cellular reprogramming was not related to LPS sensitivity, as the surface expression of TLR4 was unaltered. Moreover, M-CSF secretion increased even after stimulation with agents directly affecting post-receptor mechanisms, suggesting that the process does not involve the aberration of membrane receptors.

PU.1 is the main promoter involved in M-CSF activation [31, 32]. We found an elevation of PU.1 associated with several MΦ characteristics in MO obtained at 3 months after CPB. PU.1 is a critical regulator of MO function in the bone marrow and periphery under resting and stress conditions [31]. Under resting conditions, PU.1 supports hematopoiesis while having anti-inflammatory properties in the cells outside the bone marrow [30, 37]. It also encourages the differentiation of MO into MAC while inhibiting the emergence of DC [21, 22]. These effects are mostly achieved via M-CSF [21, 30, 37]. Interestingly, demethylation of the PU.1-encoding *SPI1* promoter was observed, and it was associated with increased expression of the protein level of PU.1. Epigenetic modulation of PU.1 activity can easily sustain the secretion of M-CSF, because the *SPI1* expression is no longer inhibited.

Though our study was not powered to look for statistical differences, we showed some increase in markers of opportunistic infection [1, 33, 34]. Elevation of CMV IgG titer was present and is most likely secondary to re-activation of latent infection not the acquisition of a new infection [7, 36]. However, small population size and heterogeneity of response caution against any conclusion but it revealed a potential direction to further develop this study. Also, the deactivation of a viral infection can be secondary to the dysfunction of other leukocyte populations, emergence of myeloid-derived suppressor cells, or may occur independently of MO dysfunction. A similar cautionary approach should be used to observed elevation of CRP suggesting smoldering inflammation and increase in atherosclerotic risk [35, 40, 41].

Experimental design of our study was aimed at increased robustness despite its pilot nature [4, 5, 36]. Utilizing CPB to study the long-term consequences of severe surgery allowed for an assessment of the pre-stress immunological make-up. We also significantly reduced the effect of high inter-individual heterogeneity inherent to the study of immune system function [42]. Despite the small sample size, the longitudinal design of this study further added to its quality. Our standardized protocol minimized hospital practice-related variations. There was > 90% post-CPB compliance with medications that can affect the immune system, including statins, aspirin, and narcotics.

However, there were several limitations as well. First, the pre-operative use of statins, tobacco, and hypo/hyperglycemic agents was frequent in our study population; these agents are known to affect the performance of the immune system under stress. Second, the small sample size limited the measurement of certain clinical outcomes, such as pneumonia and kidney failure. Third, we did not account for MO population shift in depth. The depression of T cells proliferation in response to deficient MO could account for an emergence of alternatively activated MO, myeloid-derived suppressor cells or monocyte anergy [43]. Fourth, although CPB is a good example of severe critical care stress, other clinical illnesses have distinct features which may play a post-insult role. It is unclear whether the CPB-related stress was more severe than the cardiac surgery-related, anesthesia-related stress or the observed finding are typical to any several enough insult to the immune system. Finally, despite the designed our study in longitudinal fashion it remains to be seen if the increase expression of MO-secreted M-CSF is incited by changes in methylation level of SPII, or the acquisition of these features is part of broader tendency to acquire macrophage characteristic after severe insult.

## Conclusions

In summary, we demonstrated the persistence of post-CPB MO alterations as late as 3 months after heart surgery [1, 6, 11, 12]. Second, we found a concomitant over-activation of M-CSF/PU.1 with epigenetic changes in the critical promoter region of a PU.1-encoding gene in recovering patients. The clinical significance of the study is that despite apparent recovery from heart surgery, the immunological aftermath continues for at least 3 months and potentially longer. Though our sample size was not powered to uncover the clinical correlates, we found evidence of newly-acquired immunosuppression and elevation of markers in atherosclerosis concomitant with the PU.1/MCSF activation pathway.

The next step is to characterize the persistence of post-surgical immune aberrancies beyond 3 months, investigate other epigenetic mechanisms involved in maintenance of the newly-acquired immunostasis following CPB, and selectively targeting M-CSF/PU.1 activity to see whether the recovery of pre-insult MO function can be achieved. Finally, investigating the long-term activation pattern of MO and M-CSF/PU.1 in other clinical scenarios will define if the described mechanism is specific to CPB or of more universal nature.

## Abbreviations

M-CSF: monocyte colony stimulating factor
MO: monocyte
MΦ: macrophage
SOCS: suppressor of cytokine signaling
SIRS: severe inflammatory response syndrome
CPB: cardio-pulmonary bypass
CRP: C-reactive protein
CMV: cytomegalovirus
